# Supporting parents of transgender, nonbinary, and gender-expansive youth: Considerations for health intervention development

**DOI:** 10.1016/j.outlook.2025.102677

**Published:** 2026-01-14

**Authors:** Dalmacio Dennis Flores, Jessica Webster, Alexandra Casison, Anjelique Agudo, Seul Ki Choi, Keosha Bond, Gigi McGaughey

**Affiliations:** a University of Pennsylvania School of Nursing, Philadelphia, PA; b University of Pennsylvania School of Arts and Sciences, Philadelphia, PA; c City University of New York School of Medicine, New York City, NY

**Keywords:** Transgender, LGBTQ, Nonbinary, Gender-affirming care, Parents, Qualitative

## Abstract

Parental support is associated with improved health of transgender, nonbinary and gender expansive (TNGE) youth. However, parents struggle to find support in navigating gender-affirming care with their child. Nursing plays an indispensable role in these dyads’ wellbeing. This study aims to identify barriers and intervention preferences for enhancing knowledge and group-level support for parents of TNGE adolescents. Guided by the Information-Motivation-Behavioral Skills Model, we conducted four virtual focus groups with parents (n=19) of TNGE youth. Content analysis was conducted to identify themes around parent needs. Predominantly white mothers from the PA-NJ-NY area participated. Lack of TNGE-relevant knowledge from typically trusted sources and limited resources were identified as primary informational barriers. Suggested intervention components to increase motivation/behavioral skills included education programs, support groups, and expanding resources. Findings highlight gaps in supportive systems available to parents of TNGE youth. Future research could include incorporating these intervention components.

## Introduction

Youth who are transgender, nonbinary, and gender expansive (TNGE) are individuals with gender identities or expressions that reach beyond, resist, or do not conform to culturally defined norms associated with their sex assigned at birth (Mangin, 2018). In 2023, the Centers for Disease Control and Prevention (CDC) reported that approximately 3.3% of youth identify as transgender and 2.2% are questioning their gender identity (CDC, 2023). While increased visibility and societal acceptance may encourage more youth to openly identify as lesbian, gay, bisexual, transgender, or queer (LGBTQ+), TNGE youth continue to face significant barriers to equitable health outcomes.

Existing research demonstrates TNGE youth experience higher rates of depression, anxiety, substance use, suicidal ideation, suicide attempts, and sexually transmitted infections (CDC, 2023; Durwood et al., 2017; Newcomb et al., 2020; Perez-Brumer et al., 2017, Reisner et al., 2019; Suarez et al., 2024). TNGE youth experience higher levels of psychological distress than both their cisgender, heterosexual peers, and their cisgender, sexual minority peers (Newcomb et al., 2020; Wittlin et al., 2023). Within the LGBTQ+ community, TGE youth are twice as likely to attempt suicide than cisgender youth (Perez-Brumer et al., 2017; Raifman et al., 2020). Additionally, TNGE individuals are disproportionately affected by minority stressors (e.g., transphobia, homophobia, violent victimization, unstable housing, and systemic healthcare inequities), which have also been linked to negative health (Chodzen et al., 2019; Choi et al., 2015; Johns et al., 2019; Newcomb et al., 2020; Parchem et al., 2025; Price et al., 2024; Reisner et al., 2015; Sevelius, 2013). The politicization and curbing of access to gender-affirming education and care by the current administration (Executive Order No. 14168, 2025; Executive Order No. 14187, 2025) not only increases immediate health risks but can also lead to disengagement from healthcare systems later in life, further compounding disparities (Pham et al., 2021, 2023). The adverse impact of these systemic challenges extends to parents, caregivers, and guardians (hereafter referred to as parents) of TNGE youth, who often struggle to find accurate information and affirming resources to best support their child (McKay & Fontenot, 2020).

A growing body of research underscores the critical role of family support, especially from parents, in promoting positive health outcomes among TNGE youth. Unsupportiveness—such as exclusion, limiting access to appropriate care, and insufficient knowledge specific to TNGE identities—can significantly contribute to adverse health and suicide risk for this population (Klein et al., 2023; McGregor et al., 2024; Pflugeisen et al., 2023). Conversely, family support is a primary predictor of well-being and is protective of TNGE youth mental health (Johns et al., 2018; McGregor et al., 2024; Pflugeisen et al., 2023). Higher levels of caregiver support have been associated with reduced rates of suicidal ideation and attempts, substance use, anxiety, and depression (Gower et al., 2018; McGregor et al., 2024). Nurses increasing the capacity of parents to be supportive of their TNGE patients is therefore essential to ensuring long-term health and resilience for this population.

Research specifically examining the needs of parents raising TNGE children is limited. Existing studies focus on risk factors and prioritize youth outcomes within healthcare and community contexts, with fewer exploring the challenges faced by parents. There is a dearth in qualitative research that explores parents’ needs related to adjustments in their parental self-image, informational gaps, and proffered solutions. Studies that do examine parental needs suggest significant emotional burden and a desire for education around gender identity and navigating financial and logistical aspects of the healthcare system (Pham et al., 2021; Pullen Sansfaçon et al., 2015). Previous research also describes the complexity of the parent’s role—often managing their own emotions related to acceptance, identity, and societal stigma, while simultaneously seeking reliable medical information and striving to meet their children’s needs (Alegría, 2018; Katz-Wise, Galman, et al., 2022; Katz-Wise, Gordon, et al., 2022; Tyler et al., 2025). There is a knowledge gap in parent self-care strategies and interventions to process their own internal experiences (e.g., uncertainties, uncomfortable emotions) (Alegría, 2018). Parents themselves view their own well-being as low priority and only secondary to their child’s health, but their own unmet needs may have a deleterious effect on the quality of their parenting (Warner et al., 2021).

Nurses and other clinicians are in opportune positions to address the educational and support needs of parents with TNGE youth. By capitalizing on routine wellness visits, nurses who provide care to TNGE youth may also directly work with parents to address the barriers that confound their readiness to support their child’s gender-specific health, including access to sexual health prevention options (Mehringer & Ford, 2021). Thus, our main goal is to describe parents’ experiences accessing educational and supportive resources and solicit their ideas for evidence-based, parent-focused interventions that center the emergent health questions and concerns of TNGE youth. To our knowledge, this study is among the first to directly solicit intervention suggestions and preferences from these parents, providing critical insight into this population’s needs.

To guide the development of interventions for parents, the Information-Motivation-Behavioral Skills (IMB) Model provides a valuable framework. Originally developed to explain and promote health behavior change, the IMB model emphasizes that individuals require accurate information, motivation (both personal and social), and specific behavioral skills to engage in health-promoting behaviors (Fisher & Fisher, 1992). Applied to the context of parenting TNGE youth, this model suggests that parents must first gain foundational knowledge about gender diversity and sexual health (information), develop supportive attitudes and social connections (motivation), and acquire practical skills for advocating for their child, and engaging in affirming parenting (behavioral skills).

This study aims to gain deeper insight into parents’ experiences when supporting TNGE youth, as well as their unique needs when providing TNGE-specific health support. More specifically, this study aimed to explore parents’ perspectives on developing effective and accessible resources. Understanding parental preferences is essential for designing nursing interventions that are both relevant and responsive, ultimately enhancing the support available to parents and promoting better outcomes for TNGE youth.

## Methods

### Study Procedure

This qualitative study employed semistructured, virtual focus group discussions to explore the experiences, needs, and intervention preferences of parents of TNGE youth. Parents were eligible to take part if they had a TNGE-identifying child between 12 and 20 years of age and could recall having at least one conversation with the child about health and sexuality. Parents with only cisgender children were not eligible in this study. Participants were recruited during the COVID-19 pandemic in 2021 through purposive sampling methods. Recruitment channels included gender-affirming care clinics, LGBTQ+ community organizations, online parenting forums, and targeted social media platforms. Recruitment materials emphasized the study’s focus on parent perspectives to inform intervention development. Efforts were made to recruit a demographically diverse sample of parents. Snowball sampling was also employed, with participants encouraged to share the study information with their networks. Interested parents completed a brief online screening form to confirm eligibility. Eligible participants were invited to participate in a virtual focus group conducted via Zoom and all participants provided written informed consent prior to participation. All study procedures were approved by the (University of Pennsylvania) Institutional Review Board (IRB Protocol#: 842591). Participation was voluntary, and participants received a $40 Amazon gift card as compensation for their time.

### Setting

Our research into parents’ needs and suggested solutions was concentrated in the tristate region of Pennsylvania–New Jersey–New York to underscore parental experiences in areas that in 2021 had notable access to gender-affirming services for TNGE youth and their families. Recognizing the wide disparity in available clinical services available to these dyads across the country, we focused on this geographic area as a guide for future intervention work in less-resourced areas.

### Data Collection

Four virtual focus groups were conducted with a total of 19 parents between April and November 2021. Each focus group was guided by a semistructured protocol designed to elicit participants’ perspectives on educational and support needs, barriers to affirming care, and preferences for intervention components. Each focus group session lasted 60 to 90 min and was audio-recorded. After initial introductions, participants were provided a hypothetical intervention mapping scenario, “If a generous donor gave members of this group $10 million to design a program to educate parents about the issues pertinent for their TNGE children’s sexual health, how would you use that money?” Based on their responses, follow-up probes were asked to elicit more details such as their thoughts on an individual-level vs. a group-based educational session, setting (“Should it be hosted in schools? In-person? Online?”), scheduling considerations, and potential activities TNGE youth might want to engage in as part of a parent-focused intervention. Fifteen participants took part in the first three focus groups, with similar responses observed at that point. A fourth focus group was conducted with four more parents to definitively ensure data saturation, or the point when no new information emerged.

### Reflexivity

A cisgender, gay-identifying, senior member of the team trained in qualitative approaches moderated all four focus groups. The moderator was joined by a graduate student who identifies as a queer woman and served as a note-taker. The research team’s LGBTQ+ identities and the moderator’s presence as the sole man across all focus groups may have impacted how participants engaged with the questions. Additionally, almost all of the members of the research team involved with data collection do not identify as parents, which was considered throughout the process of interpreting findings. The study member who is a parent to a lesbian young adult was assigned to be the primary coder during data analysis.

### Analysis

Focus group audio recordings were transcribed verbatim and analyzed using content analysis (Graneheim & Lundman, 2004). Manual transcription of the focus group recordings was conducted by vetted research assistants trained in qualitative techniques. Each transcription draft was verified for accuracy by a second research assistant, with attention paid to discussion flow, regional speech characteristics, and pauses. Audio recordings were deleted after verification of the transcripts. The coding team iteratively developed and refined a codebook, guided by both the IMB framework and emergent themes across the data. The analysis focused on identifying challenges and parent-driven recommendations for intervention development. We used an a priori codebook during directed content analysis to identify IMB-specific theory-based beliefs (e.g., informational barriers, potential motivating interventions, and behavioral supports) and conventional content analysis to identify other pertinent issues and emerging content (Hsieh & Shannon, 2005). A study staffer manually conducted the primary coding on an Excel spreadsheet with intermittent quality checks by the senior investigator. Coding discrepancies were discussed and resolved through consensus. Further analysis of the coded data was done to determine relationships between codes across participant responses and focus groups using the constant comparison method to identify patterns and themes (Charmaz, 2014) and subsequent data saturation (Bryman, 2016). Codes that shared overlapping meanings were merged and eventually formed the resultant themes. Exemplar quotes across focus groups were chosen for each theme to depict the rich details provided by parents.

Journal entries were completed by the moderator and research assistant after every focus group to detail their reflections. Members of the team also met after every focus group to discuss participant responses. These emerging response patterns were discussed as part of data collection and the subsequent analysis. Triangulation for confirmability comprised the multiple transcript reviews, journal entries, and team meetings. Member checking with focus group participants themselves was not employed due to the contested nature of reliably validating responses in qualitative research (e.g., fixed truth of reality, changing one’s mind about an issue, and researcher’s subjective interpretation of data) (Morse, 1994; Sandelowski, 1993). To account for credibility, findings were presented to a subsequent 14-person Community Advisory Board composed mainly of parents.

## Results

A total of 19 cisgender mothers of TNGE youth participated in four virtual focus groups. Most participants were White, with one participant identifying as Black. The mean age of index children was 17 years (SD = 2.51), ranging from 12 to 20 years, with diverse gender identities (Table 1). Seven parents had transgender sons (37%), six had transgender daughters (32%), and six had children who identified as nonbinary (32%).

Themes that emerged from analysis were organized into two broad categories: challenges to adequate knowledge and support (“barriers”) and preferred solutions to address these barriers (“solutions”).

### Barriers

#### Limited Information From Trusted Resources

Participants encountered difficulties in accessing accurate, relevant information to support their TNGE adolescents through typically trusted resources, such as school staff and healthcare providers. Concerns that consistently arose included limitations in professionals’ knowledge of and sensitivity to the unique needs of TNGE youth. Participants described that seeming limitations in healthcare provider awareness especially contributed to their own knowledge gap.

“There’s some doctors who don’t understand. I think that’s really important that the medical professionals get trained, because they can’t have proper talks. Because a lot of times kids might ask their doctors something they wouldn’t ask a parent if they’re uncomfortable talking to a parent, and when the doctors don’t understand, that makes it difficult.”

(Johanna, FG1)

“However, they have sex ed at my daughter’s school starting in 7th grade and...the coach is teaching the class and he calls the things—he calls anatomy “man parts” and “lady parts,” and he shows them YouTube videos. I said, ‘No, no.’”

(Mara, FG4)

#### Limited Access to Specialty Clinics

Multiple focus group participants identified difficulties in both locating and accessing affirming care for TNGE youth. Parents underscored the benefits of seeking affirming care but lamented long wait times at gender clinics to receive care due to limitations in staffing and resources.

“The systems were already there but not have to wait 6 months to get into them, to not have to kinda jump through hoops. Cuz once you’re in, there’s a social worker that literally jumps through all the hoops for you.”

(Margie, FG1)

#### Cisheteronormativity in Available Resources

Sexual health resources, from which parents can typically draw to support their developing adolescent’s health, were also identified as lacking adequate information relevant to TNGE youth. For example, reflecting on the heteronormative biases in sexual health resources, parents expressed the need for developing an inclusive curriculum that incorporates the unique health issues related to this population. From her experience attending a workshop for parents, one participant recalled:

“We talked about birth control, we talked about condoms, we talked about breast cancer and the surgeries that happen with breast cancer. But we were never talked about, like, there’s testosterone and this is the side effect and there’s top surgery and this is the side effect. I wish this had just been discussed in just one class. Just one class.”

(Margie, FG1)

#### Nonvisibility of LGBTQ+ Staff

Recognizing their own cisgender identities, parents expressed frustration over the lack of visibly LGBTQ+ identifying staff at healthcare facilities. Concordance between youth and provider identities was reported as ideal to help address limitations from not having a shared lived experience with their child.

“My kid really only wants to talk to providers who identify as LGBTQ themselves. So we’ve run into that with therapy, with healthcare providers.... We saw [an LGBTQ+ provider] at a gender clinic recently. That [match] helps because I think they feel like that person can speak to them in a language that they can understand and feel comfortable with.”

(Mara, FG4)

### Solutions

#### Creating TNGE-Centered Education Programs

In all four focus groups, participants identified education and support initiatives as critical for parents of TNGE youth. Foremost, participants expressed the need for future cohorts of parents to be educated early about TNGE health rather than parents themselves having to find resources to learn about the complex issues affecting this youth group.

“I think all of us have expressed how much we have learned since our kids have come out. And that’s like, over the course of years. I’m sure on some level, we wish we knew some of it sooner.”

(Jamie, FG2)

Second, parents identified the need for trusted adults, such as teachers and healthcare providers, to be better trained in TNGE health. They explained their common experience of feeling more knowledgeable about TNGE issues and preferring actual professionals to be more well-versed about these issues.

“I think we’ll probably all agree that we do a lot of educating to the people in our kids’ lives, we’re educating their educators, their teachers, their pediatrician, their dentist, the person at the lab taking their bloodwork. Like, we’re educating everyone all the time. And the more those systems are educated to support and support us, the easier it is for us.”

(Grace, FG4)

“I’m a huge advocate of educating the educators because if they don’t understand what transgender even means, let alone how to talk about sex to transgender youth... you kind of have to start with square one”

(Johanna, FG1).

Participants brainstormed content related to TNGE-specific health, sexual health, and the intricacies of parenting TNGE youth. For those looking to acquire the essential knowledge and skills to navigate parenting, participants described a “101” course, covering basic or introductory-level topics. Most participants expressed preference for a multiple-session, facilitator-led intervention that would allow a pre-established group of participants to explore a specific learning category in-depth, with each meeting focused on a relevant topic progressing from introductory to advanced knowledge in that area. Parents also expressed technology-based formats such as virtual learning and prerecorded modules as ideal to accommodate their busy working schedules and to protect parents’ identities. Table 2 details components of what participants deem as essential to cover with future cohorts of parents.

#### Developing Parent Support Networks

Participants across groups emphasized the positive impact of an affirming community’s support. Recognizing that parents who receive interpersonal support can better assist their children, they suggested interventions aimed at building connections between families of TNGE youth. Support groups were recommended as part of a parent-based intervention. Participants described creating a safe, neutral space where parents can have open-topic discussions about TNGE issues, seek advice, and address concerns.

“I’m finding with a lot of the families at our school that it seems to be the most important thing - that the parents want to talk and they wanna hear from people and parents who’ve done this already and those who are a little further ahead in the process. They wanna hear other people’s experiences and they wanna know that ultimately years down the road, that it’s all going to be okay.”

(Mara, FG4)

Relatedly, focus group participants acknowledged that for any support programs to be successful, it must include parents who are still in the early stages of learning about a child’s TNGE identity. Specifically, participants emphasized identifying parents who struggle with recognizing and supporting their TNGE child’s emerging gender identity and parents who may be struggling with acceptance. Participants discussed the need to disseminate information to parents that may be harder to reach for reasons related to religious or cultural beliefs, location, and general awareness about LGBTQ+ issues.

“It’s really hard to reach all the parents, but it is possible....and I think an idea of an overall conference is awesome for parents like us. For parents that are new to this and really struggling, I think this small groups work really well and they might be ready to go to the conference and learn a lot more.”

(Johanna, FG1)

“I think one of the problems is how do we find those parents? That seems to be the biggest issue, is how do you get that information out to parents without outing the parents, because some of them may be afraid of that? I think it’s really easy to come up with the things to give to those parents, we just have to be able to find them and make it so that...when they walk by, they don’t have to pull a little tab off the thing and out themselves”

(Ruth, FG1)

#### Increasing Availability of Resources for Clinics and TNGE Research

Participants suggested funding established gender-affirming care organizations and strengthening clinic-based services to meet the growing need for TNGE healthcare. Clinic expansion could decrease the wait time for appointments, increase the array of services offered, and provide care for a broader population of TNGE youth and families.

“Utilize the resources that are already there to provide more gender affirming care and to provide education. Give them the resources they need to really run with that.”

(Margie, FG1)

One group identified the need for enhancing clinical research as a component to support for parents of TNGE youth. Participants suggested allocating resources toward TNGE health and sexual health research, specifically as related to the medical aspects of transitioning.

“We need some more medical research for these children, because this is the first generation of kids that have been allowed to transition earlier on in life, and there’s a big lack of guidance for us parents.”

(Jill, FG3)

Proposed areas for further research included the implications of puberty suppression, medication side effects, and best practice research for parents as they navigate medical decision-making with transitioning youth.

One of the things we’ve been struggling with is long-term fertility and what to do. And I know you’re talking about adolescent age, but we’re thinking about starting hormones. And then do you do a sperm banking, or egg retrieval? It’s hard to make a decision like that at these ages, so it’s a big topic I feel like I wish there was more information, you know.

(Nadia, FG3)

## Discussion

This study adds to the call for nurses and other clinicians to enhance educational and support resources for parents with TNGE youth (McKay & Fontenot, 2020). From our study, participants identified critical barriers encountered by parents, including the lack of relevant knowledge among typically trusted sources and limited support for their families. Participants provided solutions to overcome these challenges to enhance individual knowledge and group-level support. With nurses’ ubiquitous presence throughout healthcare and family nursing research, a dynamic area of scholarship, the discipline is key to mitigating disparities long associated with the health of TNGE youth and available support for their parents (Carroll et al., 2024).

Similar to previous studies (Alegría, 2018; Dunlap et al., 2023), parents’ perception of the limited information around TNGE youth’s sexual health was discussed at length in all focus groups. Participants emphasized the value of offering diverse training modalities to accommodate differing levels of awareness, acceptance, and logistical constraints. The use of varied content formats and routes of dissemination has been successful in previous LGBTQ-focused educational interventions with parents and service providers (Lozano et al., 2022; Yu et al., 2023), suggesting these efforts will expand access to these resources if replicated. Furthermore, parents emphasized the role of social acceptance in shaping their needs, advocating for education interventions that safeguard privacy and facilitate virtual or anonymous learning—an insight particularly relevant amidst increasing legislative restrictions on TNGE individuals’ rights (Christensen et al., 2025; Kuper et al., 2022). Considering the diverse socioecological environments parents of TNGE youth are embedded in will be essential to the success of future education and support interventions. Future studies should additionally take advantage of this parent population’s collective body of knowledge (Townley & Henderson, 2024); parent engagement with community-based participatory research methods, such as co-design, will improve the effectiveness and acceptability of newly developed interventions.

Parents’ desire for enhanced education and support structures is becoming increasingly documented (Dunlap et al., 2023; Katz-Wise, Galman, et al., 2022; Katz-Wise, Gordon, et al., 2022; Lawlis et al., 2020). In cases where support groups already exist, research demonstrates the perceived positive effects of engagement on parent well-being and self-efficacy. Identifying a designated space for parents to express and process their feelings is critical to forming group-level support (Dunlap et al., 2023; Hillier & Torg, 2019; Malpas et al., 2022). However, while support groups for parents are abundant in some geographic areas, factors such as local political climate and population density can prevent them from forming in others. Therefore, virtual options such as secure teleconferencing software may be recommended by healthcare providers for parents in these environments. Future studies should pilot test virtual interventions to explore their ability to increase parent knowledge about TNGE issues and improve parent and youth mental health as these may be the most feasible option to reach those in rural or in unaccepting settings.

The IMB model provides a useful theoretical framework for nurses to conceptualize parent-suggested interventions (Fisher & Fisher, 1992). As applied in this study, the IMB model suggests that addressing parents’ information gaps, bolstering motivation through social support, and building practical skills can collectively enhance their capacity to support TNGE youth. This model aligns with the multicomponent intervention elements identified in this study and that nurses may easily address as well in their nursing practice. For example, reinforcing school nurses’ capacity to act as mediators between newly out TNGE youth and their unaccepting parents by providing informational resources can help families cohere once again as a family unit (Neiman et al., 2023). Similarly, families in transition to accommodate a child’s gender identity can benefit from an informed practitioner who is able to support parents’ complex struggles (Wagner & Armstrong, 2020). To promote parental motivation to support TNGE youth, nursing interventions must be informed by contemporary science. Previous work with parents used a pathologizing approach focused on negative emotions, parental grief, and secondary stigma (de Bres, 2022). Going forward, nursing interventions are better poised to focus instead on affirming approaches such as resiliency and pride around having TNGE children. Finally, to maximally ensure the uptake of behavioral skills, nurse researchers can advocate for intervention development that meaningfully centers end-user realities (see Katz-Wise et al., 2024 and Paglisotti et al., 2025). Beyond tokenistic community-engaged research gestures, nurse interventionists can adhere to consistent co-creation tenets.

It is critical to note that the U.S. sociopolitical climate has changed significantly since data were collected in 2021. The results of this study reflect viewpoints under Biden-era policies and legislation, which were generally more accepting and/or affirming (e.g., expanding the Affordable Care Act’s Section 1557 to provide specific nondiscrimination protections for transgender people’s coverage and quality of care) than those of Trump’s second term (Centers for Medicare and Medicaid, 2024). Due to the current anti-transgender efforts of the U.S. federal government, the future of gender-affirming care and other publicly funded resources for TNGE individuals, especially minors, is uncertain. Scientific bodies such as the American Psychological Association (APA) have established gender-affirming care as medically necessary (APA, 2024), but government actions, such as EO14168 *Defending Women From Gender Ideology Extremism and Restoring Biological Truth to the Federal Government* and EO14187 *Protecting Children from Chemical and Surgical Mutilation*, have threatened clinics’ funding and legal ability to provide these services (Executive Order No. 14168, 2025; Executive Order No. 14187, 2025). Therefore, it is unclear whether participant-suggested interventions to strengthen and expand gender-affirming care and TNGE-related research can be pursued with federal support at this time. However, nurses monitoring the legal landscape and identifying opportunities for advancement in their clinical practice and research is imperative to safeguard the health of TNGE youth and their parents during these challenging times (Phillips et al., 2025). Even as healthcare systems that once provided gender-affirming care across the country are systematically being shuttered with threats of withholding federal funding, nurses in all aspects of their practice can remain a stronghold of inclusivity (Porter et al., 2025) and hold firm to the profession’s key values of equity, inclusivity, and dignity (Bower et al., 2025).

Parents struggle to find TNGE-competent providers, and they have limited access to affirming clinics that go beyond cisheteronormative resources. Solutions proffered centered on increasing their own levels of knowledge about TNGE health and supporting their connection to affirming families and networks. Our study is one of the first to articulate components of potential nursing interventions to address the health of TNGE youth via their parents. Future research is needed to build and test nursing-centered resources and programming across the healthcare settings that these families navigate.

## Limitations and Recommendations

Aside from the gap between the time data were collected and the current issues affecting TNGE youth and their parents, this study’s findings should be considered within a few additional limitations. First, our participants were predominantly White and all identified as mothers, limiting the findings’ validity beyond this population. Ensuring racial, ethnic, gender, and socioeconomic diversity in future research samples will increase generalizability of findings to include non-White parents, fathers or other guardians, and differently resourced areas. Intersectional experiences of caregiving in marginalized racial/ethnic and socioeconomic contexts remain underexplored and could be examined in future investigations via partnerships with community health organizations or recruitment via online TNGE communities. Participants were recruited mostly via gender-affirming clinics and LGBTQ+ networks, which may have excluded parents less connected to supportive communities—arguably those most in need of support. It is therefore critical that further studies attempt to reach parents in these less inclusive environments, such as through recruitment at local schools or (where feasible) places of worship, as our findings may not be transferable to these contexts. Additionally, the qualitative nature of focus groups may have led to social desirability bias, where participants shared more socially acceptable views on gender diversity and parenting. Quantitative data collection techniques, such as via anonymous surveys, that do not require prolonged and deep interactions with study team members, could mitigate this bias in future research.

## Conclusion

This study underscores the critical role of equipping caregivers of TNGE youth with accessible, affirming, and evidence-based support. A parent-informed intervention model that is multitiered, flexible, and community-embedded can address the multitude of parents’ needs—ultimately improving the well-being and healthcare access of TNGE youth and their parents. Future work should focus on incorporating and testing these parent-informed intervention components, assessing their impact on youth, parents, and family-unit outcomes.

## Figures and Tables

**Table 1:** Parents’ Participant Profiles

Parent Pseudonym	Child’s Gender Identity	Child Age

*Focus Group 1–4/24/2021*		
Christa	Nonbinary	18
Jo	Transgender son	20
Johanna	Transgender son	20
Lisa	Transgender son	20
Margie	Nonbinary	15
Ruth	Nonbinary	17
*Focus Group 2–10/6/21*		
Jamie	Transgender daughter	14
Kristi	Transgender daughter	20
Sarah	Nonbinary	15
Simone	Transgender daughter	19
Victoria	Nonbinary	14
*Focus Group 3–11/10/21*		
AB	Transgender son	17
Jill	Transgender daughter	15
Kris	Transgender daughter	20
Nadia	Transgender daughter	19
*Focus Group 4–11/15/21*		
Grace	Transgender son	17
Mara	Transgender son	16
Michele	Nonbinary	12
Nina	Transgender son	15

**Table 2:** Recommended Components of an Educational and Support Intervention for Parents

Components	Exemplar Quote

1. Supporting emerging sexual orientation and gender identity	*This [parental resource] needs to start somewhere around puberty, when kids are just starting to realize they might be different or “they might be this, they might be that.” My children went to girls’ school, so I only know that sex ed program... We need to get some information, to take with us as they’re going on their journey... (Christa, FG1)* *Walk families through a curriculum or a 101... through the details of sexuality and gender identity straight through... You need that foundation in order to talk about the sexual health stuff. Because if you don’t know the basics of who you are and what it means to be part of that community, then all the nitty gritty embarrassing sexual health stuff is way too far advanced. (Jamie, FG2)*
2. Understanding the impact of parental support on health outcomes for TNGE youth	*I think at the end of the day, parents that don’t accept their children are scared...and I think that a lot of their dismissiveness comes from fear...You can be afraid for your child, but your lack of support can lead to these very outcomes that you’re probably afraid of. Give parents the understanding of what happens to trans and gender diverse individuals who aren’t supported and what those outcomes look like, and how different it is for kids that do feel supported. (Simone, FG2)*
3. Creating supportive parent networks	*Literally, have some sort of class that’s labelled LGBTQ+ 101. “Let’s do the breakdown for you - by the attendees. You see that you’re not gonna be alone. You can ask questions and you’re there to learn.” It all goes back to education by the attendees...I think once it [parenting a trans child] affects you and you wanna learn, then those questions of doubt doesn’t even cross your mind as much. (Victoria, FG2)* *Peer groups and support groups let the parents know that they’re not alone in this and that other people have come to this situation and had very similar reactions to what they have. And that those things are all okay. “Here’s how we handle them” and “Here’s how we best support our kiddos,” no matter what they’re going through. (Simone, FG2).*
4. Using inclusive language	*You don’t want to offend your child, especially when you learn their pronouns [during] the adjustment period. It will cause so much conflict (Victoria, FG2).*
5. Emphasizing broad safety concerns	*My son is aware that there’s a lot of violence against the trans community. He’s fully aware that not everybody is as open as his support system. Which is good, because I don’t want him to go out there and get hurt. That’s definitely a big issue [to cover], safety for the trans community. (AB, FG3)* *I would echo that [safety] that’s the top priority, right? Making sure everyone knows what’s going on (Nadia, FG3)*
6. Navigating dating and identity disclosure	*I have a trans daughter so my conversations have to be a little bit different because that’s my biggest fear about all sexual experiences with her. At what point do you tell a partner, tell a date... where does that conversation start? When does it come in? (Jill, FG3)* *Make sure that everyone is aware [of gender identity]. If my son is going on a date, I don’t want his date to be startled that it’s not who they were expecting. (AB, FG3)*
